# Bacteria Community Inhabiting *Heterobasidion* Fruiting Body and Associated Wood of Different Decay Classes

**DOI:** 10.3389/fmicb.2022.864619

**Published:** 2022-05-03

**Authors:** Wenzi Ren, Reijo Penttilä, Risto Kasanen, Fred O. Asiegbu

**Affiliations:** ^1^Department of Forest Sciences, University of Helsinki, Helsinki, Finland; ^2^Natural Resources Institute of Finland (Luke), Helsinki, Finland

**Keywords:** wood decay process, bacteria community, *Heterobasidion*, fruiting body, network analysis

## Abstract

The microbiome of *Heterobasidion*-induced wood decay of living trees has been previously studied; however, less is known about the bacteria biota of its perennial fruiting body and the adhering wood tissue. In this study, we investigated the bacteria biota of the *Heterobasidion* fruiting body and its adhering deadwood. Out of 7,462 operational taxonomic units (OTUs), about 5,918 OTUs were obtained from the fruiting body and 5,469 OTUs were obtained from the associated dead wood. Interestingly, an average of 52.6% of bacteria biota in the fruiting body was shared with the associated dead wood. The overall and unique OTUs had trends of decreasing from decay classes 1 to 3 but increasing in decay class 4. The fruiting body had the highest overall and unique OTUs number in the fourth decay class, whereas wood had the highest OTU in decay class 1. *Sphingomonas* spp. was significantly higher in the fruiting body, and phylum Firmicutes was more dominant in wood tissue. The FAPROTAX functional structure analysis revealed nutrition, energy, degradation, and plant-pathogen-related functions of the communities. Our results also showed that bacteria communities in both substrates experienced a process of a new community reconstruction through the various decay stages. The process was not synchronic in the two substrates, but the community structures and functions were well-differentiated in the final decay class. The bacteria community was highly dynamic; the microbiota activeness, community stability, and functions changed with the decay process. The third decay class was an important turning point for community restructuring. Host properties, environmental factors, and microbial interactions jointly influenced the final community structure. Bacteria community in the fruiting body attached to the living standing tree was suppressed compared with those associated with dead wood. Bacteria appear to spread from wood tissue of the standing living tree to the fruiting body, but after the tree is killed, bacteria moved from fruiting body to wood. It is most likely that some of the resident endophytic bacteria within the fruiting body are either parasitic, depending on it for their nutrition, or are mutualistic symbionts.

## Introduction

The genus *Heterobasidion* within the phylum Basidiomycota contains 13 species with saprotrophic and necrotrophic lifestyles. The species complex, *H. annosum s.l.*, has a necrotrophic lifestyle and contains five species, namely, *H. abietinum, H. annosum sensu stricto* (s.s.), *H. irregulare, H. parviporum*, and *H. occidentale* (Ioos et al., 2019). Among these, *H. annosum* and *H. parviporum* are the most severe root rot disease causal agents, posing a major threat to conifer forests of the northern hemisphere (Asiegbu et al., 2005). The primary infection of *Heterobasidion* is mediated by basidiospores dispersal and germination on wounds or exposed stump surfaces. The secondary infection continuously spreads the disease to the adjacent healthy tree by root-to-root contact (Korhonen and Stenlid, 1998; Zaluma et al., 2019). The current control methods include silvicultural, chemical, and biological control approaches (Mesanza et al., 2016). Chemical and biological control methods are the most efficient (Pellicciaro et al., 2021). However, the application of chemical treatment such as urea is restricted in Europe due to its potential negative impacts on the environment (EU Reg. 2020/1160). In contrast, the persistent use of the biocontrol product (e.g., Rotstop), which is based on a single natural isolate of *Phlebiopsis gigantea*, might increase the ecological risks of pathogen tolerance (GŽibovska, 2016). Exploring other alternative efficient control approaches is therefore required.

Fruiting body is a highly nutritious multicellular structure containing spores of fungi and other microbes (Maurice et al., 2021). Its morphology, sporulation period, and longevity vary in different species (Moore et al., 2008). *Heterobasidion* species tend to be active when the temperature is above 5°C, and the spore deposition rate for each square meter can be over 1,000 basidiospores per hour (Gonthier et al., 2005; Müller et al., 2014). The spreading distance could reach 1,000 m (Möykkynen et al., 1997). It has been observed that spruce logs left on peat soil form the most abundant fruiting body (Gaitnieks et al., 2021), cull lumbar piece was also found to be significant to the fruiting body formation (Müller et al., 2007). The fruiting body provides habitat or food resources for diverse organisms, including bacteria and fungi (Elliott et al., 2019; Legzdina et al., 2021; Maurice et al., 2021). The interactions between *Heterobasidion* and associated organisms influence the development of each other and will produce a dynamic counterbalance (Deveau et al., 2018). The fruiting body associated with wood often has biochemical mechanism to defend itself against external injuries, however, with the progression of the decay process, biochemical compounds fade away, and the physical structure of fruiting body changes (Venkatesh and Keller, 2019; Legzdina et al., 2021; Maurice et al., 2021). These physical-chemical property variations influence the microbial interactions inside the fruiting body (Gohar et al., 2020). Thus, the microbiome composition of fruiting body might be of relevance for *Heterobasidion* primary infection. Despite the potential importance of fruiting body microbiome in the ecological interactions (Gohar et al., 2020; Maurice et al., 2021), almost no information is available on *Heterobasidion* fruiting body.

In contrast, deadwood is a major substrate for most wood-inhabiting fungi, and the associated fungal fruiting bodies play an important ecological role in the ecosystem. Deadwood is important for nutrient cycling, carbon sequestration, soil property, and biodiversity (Brunner and Kimmins, 2003; Litton et al., 2007; Rondeux and Sanchez, 2010; Błońska et al., 2017). Both quality and quantity of deadwood have impact on its ecological roles (Müller and Bütler, 2010). Deadwood might be also an incubator for forest diseases, since pathogens with dual lifestyles are known to survive over decades on deadwood by aid of their saprotrophic feeding (Cleary et al., 2013). *Heterobasidion* has been reported to remain in deadwood and retain infectious activity for over 62 years after clear cutting. Also, the *Heterobasidion* fruiting body can develop on 25 to 45-year-old rotted spruce wood (Piri, 1996; Gaitnieks et al., 2021). Deadwood volume, property, and mechanisms for its decay are all vital parameters for forest management (Müller and Bütler, 2010). A number of recent studies have reported on *Heterobasidion*'s decay impact on trees, the tree resistance against *Heterobasidion*, the microbiome associated with the pathosystem but not much on the fruiting body microbiome (Puentes Rodriguez et al., 2013; Keilhofer et al., 2018; Probst et al., 2018; Ren et al., 2019; Pellicciaro et al., 2021).

The bacteria-fungi or fruiting body interactions (BFIs) could be beneficial, neutral, or detrimental to each other (Wargo and Hogan, 2006). The symbionts could be obligate or facultative. The obligate symbiotic bacteria are usually beneficial to its fungal host, as they are highly dependent, while the facultative bacteria have more diverse influences. Endosymbiont usually has a positive effect, while the ectosymbiont's effects on hosts are diverse (Bastías et al., 2020).

*Bacillus subtilis*, a soil-dwelling bacterium, can form a biofilm to attach on the fungus surface (Kjeldgaard et al., 2019). Bacterium *Pseudomonas fluorescens* was recorded to stimulate both colonization and growth of the fungus *Laccaria bicolor* (Deveau et al., 2007). In a mutual interaction, bacteria consume metabolites from the fungus and contribute amino acids in return (Lackner et al., 2011) or benefit of carbon and nitrogen exchange (Sharmin et al., 2018). The symbiont could also help the fungus (Guo et al., 2017) or bacteria (Ruiz-Lozano and Bonfante, 2000) to establish or facilitate invasion to other fungi (Spraker et al., 2016) or plant tissues (Ruiz-Lozano and Bonfante, 2000). At the other extreme, the BFIs could also suppress each other. Strain GB 4-2 from the genus *Streptomyces* was reported to suppress *Heterobasidion* infection through promoting plant resistance (Lehr et al., 2008). However, some bacteria genera have an opportunistic influence on fungal pathogenicity (e.g., *Pseudomonas* spp.) (GŽibovska, 2016; Lipps and Samac, 2021; Pellicciaro et al., 2021).

Bacteria-fungi or fruiting body interactions can also increase host's tolerance to stress and promote nutrient uptake as well as plant growth (Steffan et al., 2020; Asiegbu and Kovalchuk, 2021).

The structure of microbial community in this pathosystem might result as a consequence of tripartite (bacteria-fruiting body-plant) overall interactions. Understanding the structure of the community is therefore the crucial foundation to unravel the functional mechanism of the interacting partners. Other than their direct and indirect effect on each other, many other factors also contribute to the microbial structure formation. Soil properties (Pent et al., 2017), host genetics (Maurice et al., 2021), host physical and chemical properties (Maurice et al., 2021), and microclimate (Fravolini et al., 2016) have been reported to have profound influence on the microbial structure. Besides, being different from dead wood, the appearance of fruiting body may already indicate a specific environment background. For example, moisture and temperature were recorded to have a significant impact on the fungus sporulation (Moore et al., 2008). The microbes inhabiting the fruiting body could have a smaller range compared with those in dead wood. The aims of this study were to unravel (1) the bacteria biota of fruiting body and associated dead wood of different decay classes, (2) their possible nature and functional characteristics, and (3) using the network model to unravel their potential interactions among each other, as well as possible impact on *Heterobasidion* pathogenesis.

## Materials and Methods

### Sampling

In each sampling spot, *Heterobasidion* fruiting body and the associated wood were collected; the dead wood was classified to decay classes 1 to 4 based on how deep the knife was able to penetrate the wood (Mäkinen et al., 2006). Samples were collected from a total of 38 spots. Notably, 12 of the fruiting bodies were from wood classified as decay class 1, i.e., recently dead wood; eight from decay class 2, weakly decayed; five from decay class 3, medium decayed; and 13 from decay class 4, highly decayed (decay classes represented as D1F–D4F for fruiting body and D1W–D4W for wood). All samples were collected from six Norway spruce dominated boreal forest sites in Uusimaa region, located in Viikki (Helsinki) and Myrskylä, two sites from Lapinjärvi, and two sites from Sipoo. Forest types under different management purposes were also considered in the sampling. Viikki forest site is mainly used for recreational purpose; sites from Sipoo and one site from Lapinjärvi are nature conservation areas, with or without minor human interference; and the other sites from Lapinjärvi and Myrskylä forest are managed sites.

The fruiting body and associated dead wood samples were collected from stumps, logs, and standing tree wood. Wood tissue immediately below and attached to the basidiocarp was collected, with the aid of a sharp knife and an ax if necessary. The stand age of sampling sites ranged from 0 to 109 years, the canopy cover ranged from 0% to over 71%. The elevation of sites ranged from 7.42 to 102.67 m above the sea level. The diameter of selected wood ranged from 9 to 40 cm for managed forest and from 15 to 40 cm for unmanaged forest. Stand age and canopy cover of each spot were obtained from National Land Survey of Finland (Supplementary Table 1; https://kartta.paikkatietoikkuna.fi/). Samples were stored in a cooling box before storage in −20°C. Sample collections were conducted in June 2020.

A combination of visual morphological identification and ITS-based analysis was used to confirm that the collected fruiting bodies were *Heterobasidion* spp., and that the associated deadwood was infected by it.

### DNA Extraction, Amplification of 16S rDNA Region, and Sequencing

All samples were prewashed over running water followed by immersion in 70% ethanol (EtOH) for 1 min and 1% commercial bleach for 5 min and rinsed several times with sterile distilled water in order to remove surface adhering microbes. Samples were dried with paper towel and air-dried before homogenization (Moreno et al., 2014). To avoid bias, subsamples from the fruiting body were collected from four directions as well as the middle part, whereas the entire wood sample was fully homogenized. Samples were extracted following a standard cetyltrimethylammonium bromide (CTAB) method (Chang et al., 1993) with modifications (Terhonen et al., 2011). The concentration and purity of DNA were measured using the NanoDrop ND-1000 spectrophotometer (Thermo Fisher Scientific, USA). PCR amplification of the bacterial 16S rDNA V3–V4 region and sequencing were performed at Novogene (Cambridge Science Park, UK). The PCR products were purified and sequenced with the Illumina NovaSeq platform. The amplification primers were 341F 5′-CCTAYGGGRBGCASCAG-3′ and 806R 5′-GGACTACNNGGGTATCTAAT-3′. Approximately 250 nucleotides per sequencing reads were produced. Raw sequences obtained in this study are available from the Sequence Read Archive (SRA) of National Center for Biotechnology Information (NCBI) under project number PRJNA800074.

### Data Processing

The raw 16S rRNA sequences were preprocessed at Novogene, UK. The paired-end reads were merged using FLASH Version 1.2.7 (Magoč and Salzberg, 2011); the pair-end reads were spliced when reads overlapped with the same fragment. The sequence data were filtered by Qiime pipeline (Caporaso et al., 2010) to get high-quality sequences. Chimera was detected by comparing to Gold database with the UCHIME algorithm (Haas et al., 2011) and removed using the UPARSE software (Edgar, 2013). The preprocessed data were analyzed and grouped into operational taxonomic units (OTUs) using UPARSE. Sequences with ≥97% similarity were assigned to the same OTUs (Wang et al., 2007). Representative sequence for each OTU was screened for further annotation. OTUs were assigned to taxonomic groups using the Mothur software by performing sequence against the SSUrRNA database of SILVA Database (Quast et al., 2013) (taxonomic rank threshold: 0.8–1). Normalization was conducted using a standard of sequence number corresponding to the sample with the least sequences. The subsequent analysis was performed based on this normalized data. Chao1 (Chao, 1984), Shannon diversity index (H) (Shannon and Weaver, 1949), and Shannon evenness index (SEI) (Heip, 1974) were chosen to represent community species richness, diversity, and evenness. One-way ANOVA tests were conducted to identify the differences in community richness, diversity, evenness, and abundance of taxonomic groups. Principal coordinates analysis (PCoA) was used to visualize the bacterial community structure with weighted unifrac distance matrix using relative abundance of OTUs calculated by the QIIME software (Version 1.7.0). PCoA was performed by vegan package and ggplot2 package in the R software (Version 2.15.3). Functional annotation of prokaryotic taxa (FAPROTAX) database was used to identify bacterial function *via* the annotation of 16S sequence classification (Louca et al., 2016; Sansupa et al., 2021). Linear discriminant analysis (LDA) effect size (LEfSe) and one-way ANOVA tests were used to identify bacterial taxonomic and functional groups difference between different decay class samples (Segata et al., 2011). We implemented microbiome ecological network (de Vries et al., 2018) by calculating the spearman correlation of OTUs abundance among different groups through psych package in R and visualized the result by Gephi (Version 0.9.2).

## Results

### Information on the NovaSeq PE250 Sequencing

There were 6,198,431 high-quality sequences in total after denoising and quality filtering. After filtering out unclassified sequences, singletons, and sequences assigned to plant and animal specific sequences, a core set of 5,921,960 sequences was obtained. Due to the technical problem of PCR amplification and sequencing, eight samples with low read numbers were excluded; the remaining 68 samples were used for further analysis. Details on the deleted samples are stated in Supplementary Table 1. The remaining samples had sequence numbers ranging from 49,011 to 114,758 with the average number as 87,088 ± 12,577 (mean ± standard deviation). A random normalization was conducted based on the smallest sample size of 49,011 sequences among all samples and was used in the further analysis. All samples had over 99% of average Good's coverage. The rarefaction curves in Supplementary Figure 1 showed the sequencing depth.

### Bacterial Community Diversity of Fruiting Body and Wood in the Four Decay Classes

**Figure 1:**
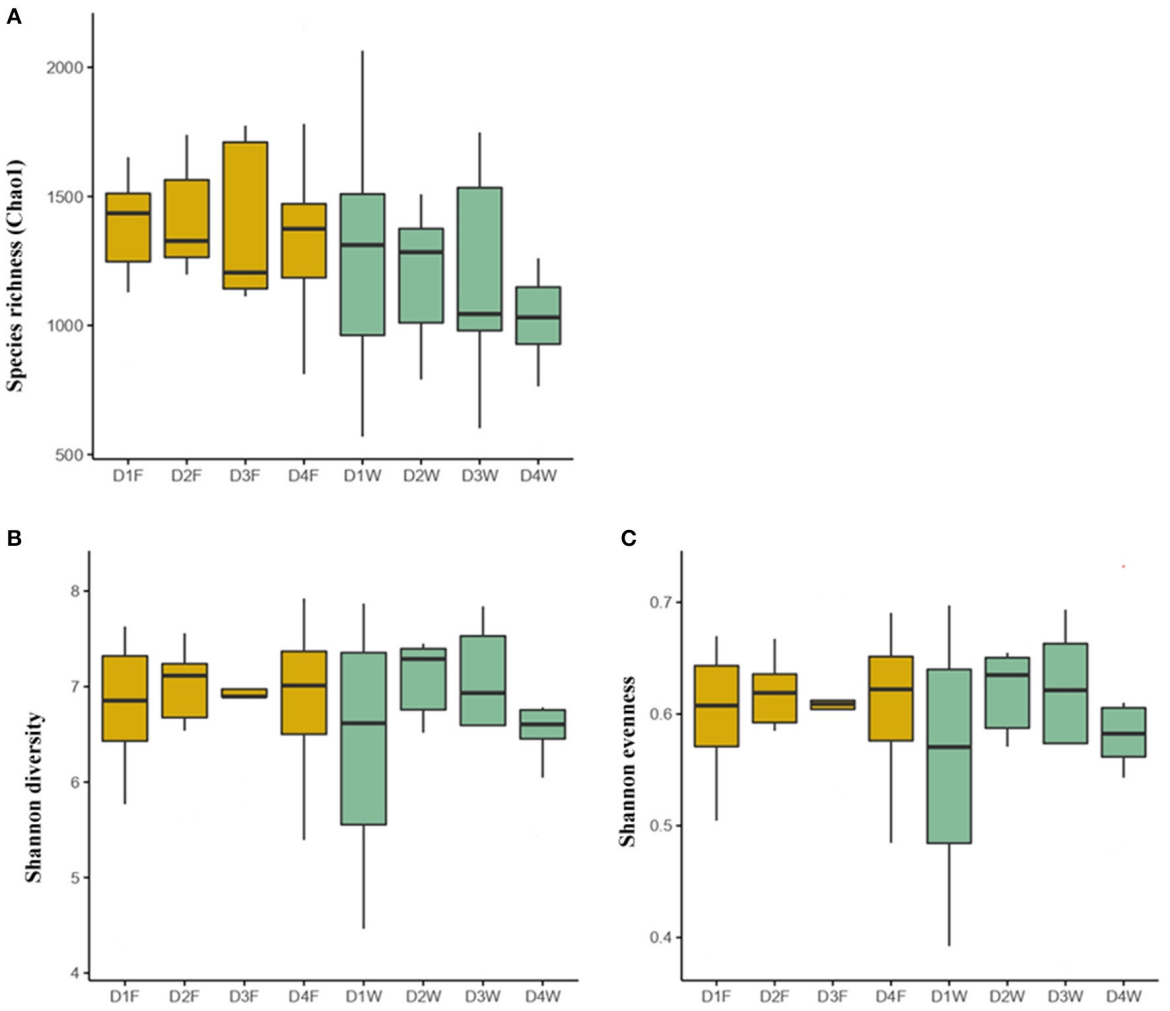
The bacterial community indices in the fruiting body and wood of different decay classes. **(A)** Species richness, **(B)** Shannon diversity, and **(C)** Shannon evenness. The outliers were removed. D1F and D1W refer to the fruiting body and wood from the first decay class; D2F and D2W refer to the fruiting body and wood from the second decay class; D3F and D3W refer to the fruiting body and wood from the third decay class; D4F and D4W refer to the fruiting body and wood from the fourth decay class.

The bacterial species richness (Chao1), diversity (Shannon diversity), and evenness (Shannon even) did not differ neither among the fruiting body and dead wood in the four decay classes nor between fruiting body and wood under the same decay class (*P* > 0.05; Figure 1).

### Bacterial Community Structure on OTU Level

**Figure 2:**
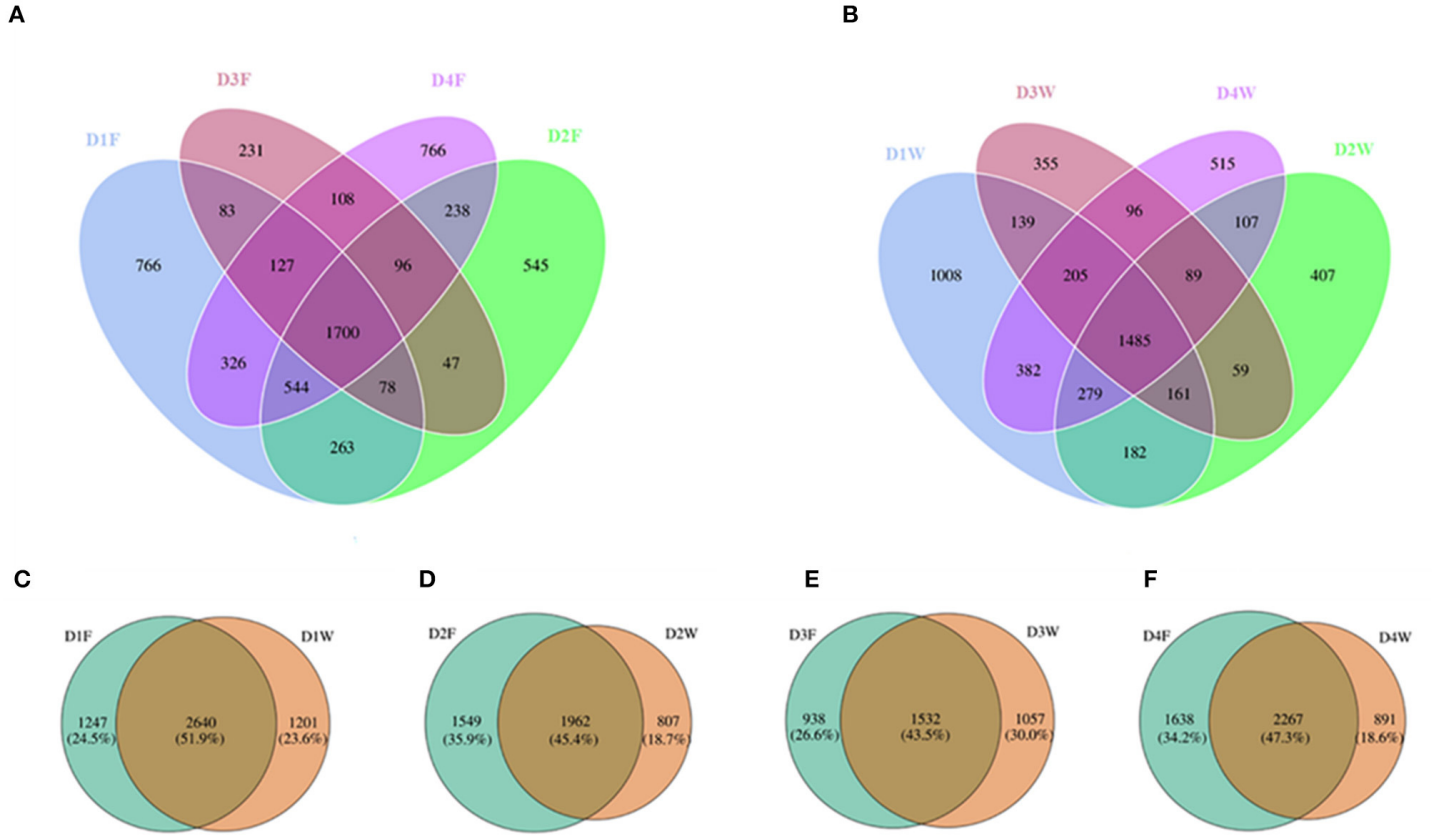
Venn diagram showing the unique and shared operational taxonomic units (OTUs) among different decay classes from **(A)** fruiting body, **(B)** dead wood, between fruiting body and dead wood from decay classes 1 **(C)**, 2 **(D)**, 3 **(E)**, and 4 **(F)**.

Across all samples, a total of 7,462 OTUs were obtained, where 5,918 OTUs were obtained from fruiting body and 5,469 OTUs were obtained from wood, 52.6% of all OTUs was shared between the two substrates. The Venn diagram revealed that the overall, common, and unique OTUs from both substrates had a varying trend of high abundance in decay class 1 with decreases in decay classes 2 and 3, and high again in decay class 4 (Figures 2A,B).

When comparing OTU numbers between wood and fruiting body in the same decay class, decay class 1 showed the highest proportion of OTUs that were common to both fruiting body and wood, and decay class 3 showed the lowest (Figures 2C,E). Wood had the maximum overall and unique OTUs in D1W and minimum in D3W and D2W (Figures 2C–E). The fruiting body had the maximum overall and unique OTUs in D4F and minimum in D3F (Figures 2E,F). In the last decay class, the fruiting body had 391 more OTUs compared with those in the initial decay stages, while wood had 310 less.

**Figure 3:**
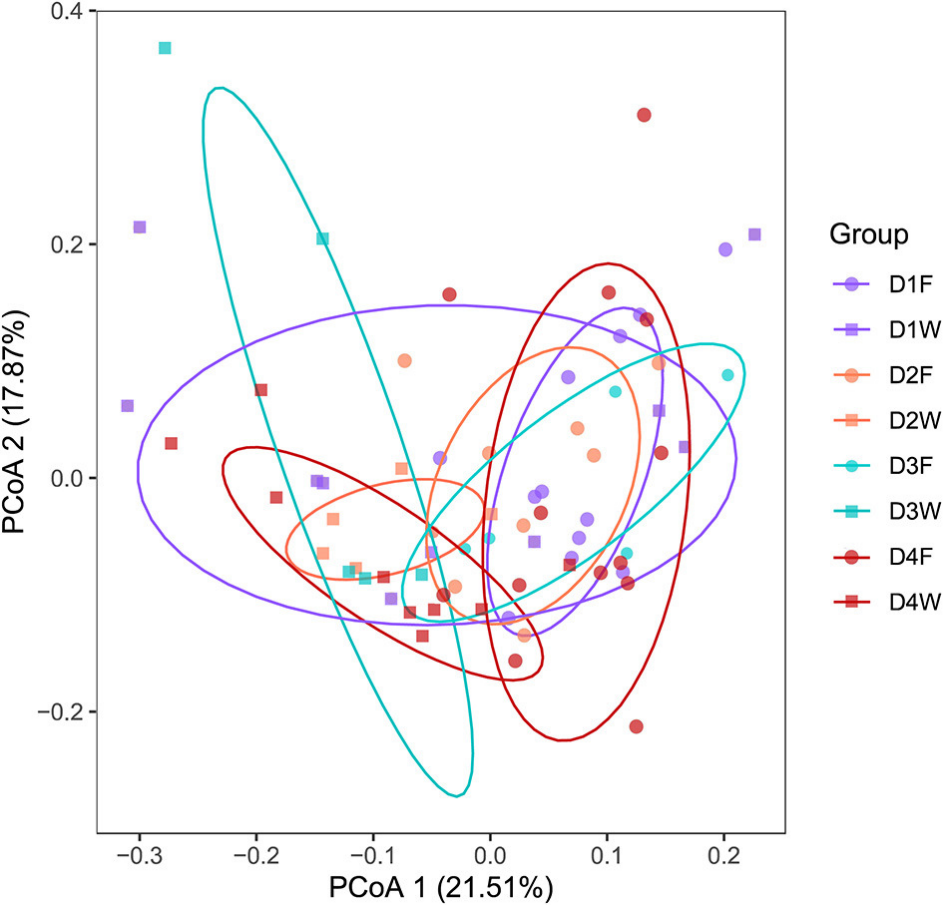
Principal-coordinate analysis (PCoA) showing the bacterial community structures of fruiting body and wood from four decay classes.

Principal coordinates analysis based on OTUs data explained 39.4% of the variation, the clusters were distinct between fruiting body and dead wood in all decay class except decay class 1 (Figure 3). The subsequent PERMANOVA confirmed the significant differences of all classes (*P* < 0.05). However, the differences were not significant among decay classes in the same substrate (Supplementary Figures 2, 3; *P* > 0.05).

In the top 50 most abundant OTUs, several OTUs contributed to the significant differences between fruiting body and dead wood in decay classes 1, 3, and 4. In classes 1 and 3, the significant patterns were higher bacteria abundance in fruiting body than dead wood. In decay class 4, both substrates had two significant abundant OTUs (Table 1A).

**Table 1A:** The most abundant operational taxonomic units (OTUs) in top 50 showing significant different patterns between fruiting body (F) and wood (W) in each decay class.

**Decay class**	**OTUs**	**Taxonomy**	***P***	**Abundance pattern**
1	OTU00047	*Labrys sp*.	0.0089	F > W
2	-	*-*	-	-
3	OTU00021	*Edaphobacter sp*.	0.0036	F > W
	OTU00007	*Sphingomonas sp*.	0.0007	F > W
	OTU02337	*Mycobacterium sp*.	0.0203	F > W
	OTU00061	*Pseudomonas viridiflava*	0.0361	F > W
4	OTU00058	*Inquilinus sp*.	0.0008	F > W
	OTU00026	*Roseiarcus sp*.	0.0011	F < W
	OTU00006	Rhizobiaceae	0.0069	F > W
	OTU00057	*Acidibacter sp*.	0.0477	F < W

Considering the same substrate, only the third decay class had significantly higher abundant OTUs than the other decay classes in the fruiting body, while in dead wood, all decay classes except class 1 had significant abundant OTUs (Table 1B).

**Table 1B:** The most abundant OTUs in top 50 showing significant different patterns among four decay classes (D) from fruiting body (F) and wood (W).

**Substrate**	**Abundance pattern**	***P***	**OTUs**	**Taxonomy**
Fruiting body	D3F > D1F	0.0202		
	D3F > D4F	0.0014	OTU00007	*Sphingomonas sp*.
	D3F > D2F	0.0134		
	D3F > D1F	0.0146	OTU00061	*Pseudomonas*
	D3F > D4F	0.0195		*viridiflava*
	D3F > D2F	0.0089	OTU00021	*Edaphobacter sp*.
	D3F > D4F	0.0360	OTU02337	*Mycobacterium sp*.
Wood	D2W > D4W	0.0361	OTU02338	*Sphingomonas sp*.
	D2W > D4W	0.0330	OTU00008	Microbacteriaceae
	D3W > D1W	0.0131	OTU00013	*Acidibacter sp*.
	D3W > D4W	0.0497		
	D3W > D1W	0.0475	OTU00033	*Staphylococcus sp*.
	D4W > D2W	0.0066	OTU00026	*Roseiarcus sp*.
	D4W > D1W	0.0016		
	D4W > D1W	0.0429	OTU00015	*Acidiphilium sp*.

### Bacterial Community Structure on Taxonomic Level

**Figure 4:**
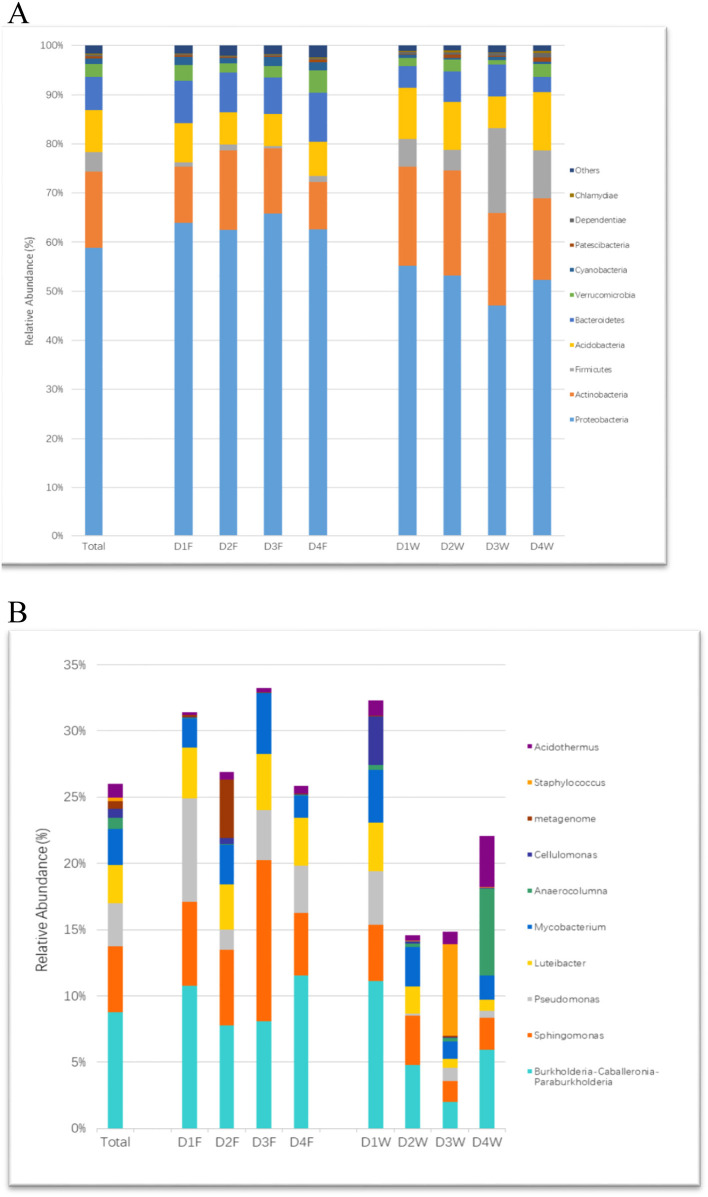
The relative abundance of bacterial community at different taxonomic levels in study groups. **(A)** Phylum and **(B)** genus.

Sequences were classified to 34 phyla, Proteobacteria was the most dominant of all groups, followed by Actinobacteria, Acidobacteria, Bacteroidetes, and Firmicutes, where they accounted for over 93.7% of the total sequences. The changing patterns of relative abundance in both substrates were the same in phyla Actinobacteria, Acidobacteria, and Cyanobacteria, while phyla Firmicutes and Bacteroidetes had the opposite trend (Figure 4A).

In the fruiting body, *Burkholderia, Sphingomonas*, and *Pseudommonas* were the most abundant identified genera. The genus *Luteibacter* had stable abundance associated with all decay classes of the fruiting body. In wood tissue, each genus had quite balanced proportions in the first decay stage, but the proportion of most genera reduced with prolonged decay (Figure 4B).

The Genus *Staphylococcus* had the highest relative abundance in D3W and was significantly higher than D1F, D4F, and D1W among all the group comparisons (Figure 4B; *P* < 0.05). The genus *Sphingomonas* was significantly higher in D3F than D1W–D4W (Figure 4B; *P* < 0.05).

**Figure 5:**
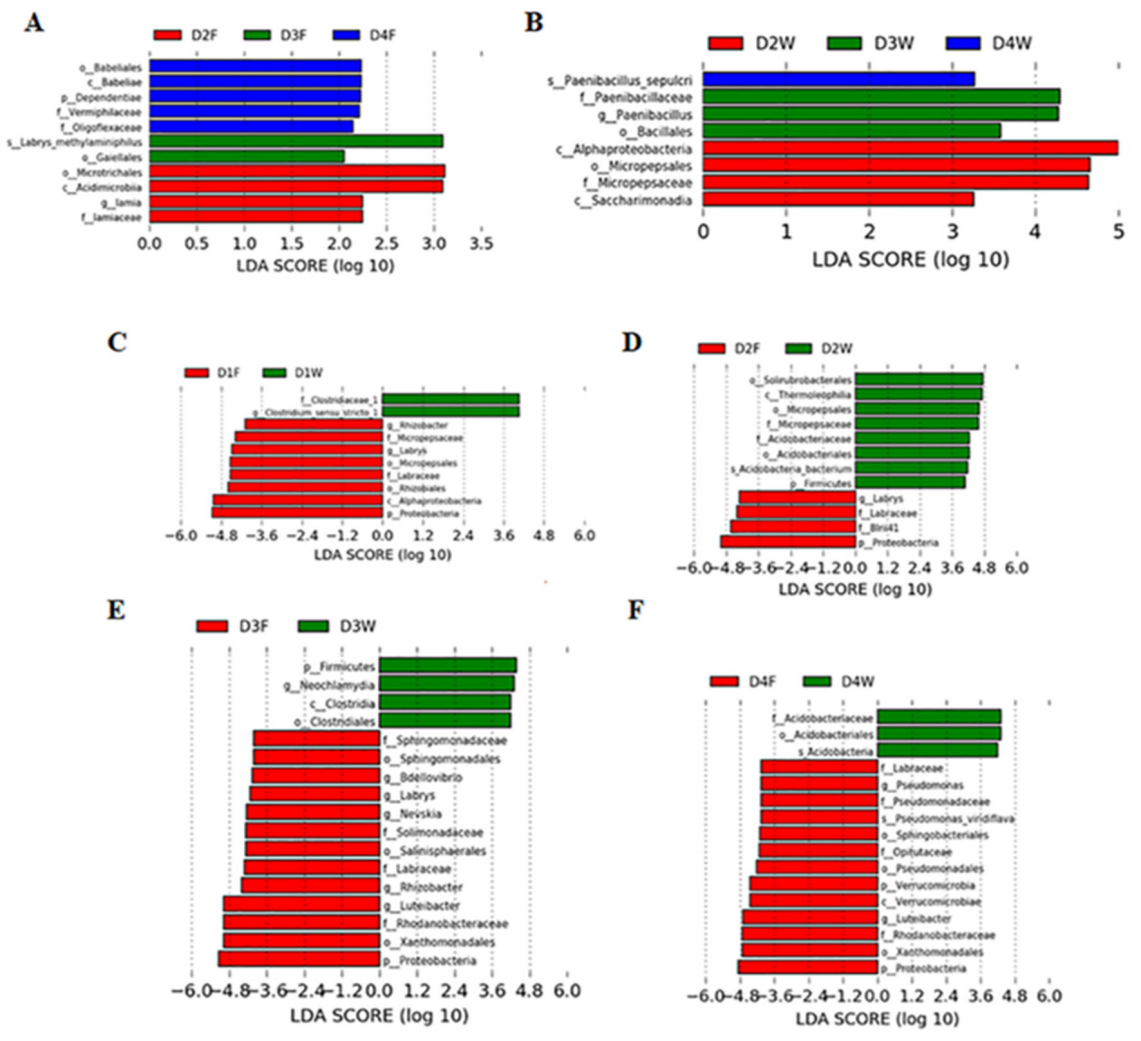
Linear discriminant analysis (LDA) effect size (LEfSe) showing bacterial phyla, class, order, family, genus, and species, which were significantly different in **(A)** fruiting body, **(B)** wood under different decay classes, and between fruiting body and wood in the same decay class, **(C)** class 1, **(D)** class 2, **(E)** class 3, and **(F)** class 4. p, phylum; c, class; o, order; f, family; g, genus; s, species.

Linear discriminant analysis effect size revealed that decay classes 2, 3, and 4 had significant higher taxonomic groups in the fruiting body and wood. In the fruiting body, decay class 4 had more abundant taxonomies, but wood had more in decay class 2 (LDA > 2.0, *P* < 0.05; Figures 5A,B). Similar differences also existed between both substrates in all classes (LDA > 4.0, *P* < 0.05; Figures 5C–F).

### Potential Bacterial Functional Structure Analysis

The top 252 OTUs, which cover 80.0% of all sequences, were assigned to 16 functional groups in FAPROTAX analysis. In total, 251 records (37.9%) were assigned to at least one group (Li et al., 2021). Chemoheterotrophy (42.2%) and aerobic chemoheterotrophy (41.4%) were the most abundant functions and were involved in carbon cycle, followed by nitrogen fixation (7.3%) and ureolysis (2.2%), which were involved in N cycle, together with fermentation (1.4%), cellulolysis (1.1%), non-photosynthetic Cyanobacteria (1.0%), plant pathogen related (0.8%), and hydrocarbon degradation (0.2%). The detailed information is found in Supplementary Table 2.

There was no functional group differences among bacteria biota observed among the decay classes in wood samples, however, in the fruiting body, phototrophy showed a pattern of D4F < D2F (*P* < 0.05); plant pathogen-related function was higher in D3F compared with that in D1F and D4F (*P* < 0.05).

**Figure 6:**
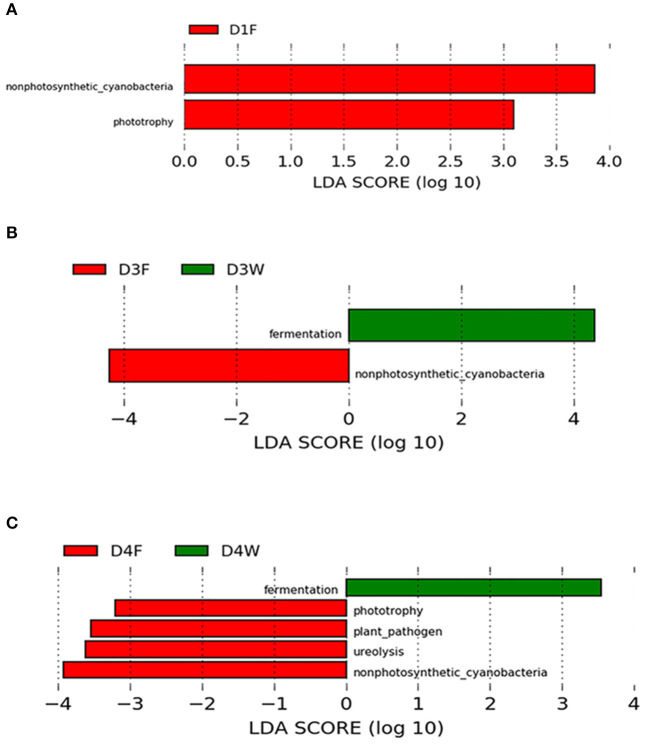
LEfSe (LDA > 3.0, *P* < 0.05) showing the predicted functional groups significantly abundant in decay classes 1 **(A)**, 3 **(B)**, and 4 **(C)**.

Comparing between wood and fruiting body in the same decay class, classes 1, 3, and 4 had significant abundant functional groups (LDA > 3.0, *P* < 0.05; Figure 6), and they changed with progress of decay. The fruiting body had more diverse abundant functional groups, while dead wood only had fermentation group in the third and fourth classes (Figure 6).

### Predicted Bacterial Interaction Analysis

The complex microbial network was unraveled by the nature of its topology characteristics (Blondel et al., 2008; Paranyushkin, 2012a). The most important topology parameters of connectivity, modularity, and Po/Ne ratio of the communities' network were selected and used to explain its activeness (Hartman et al., 2018), function-related modularization (Newman, 2006), and stability (de Vries et al., 2018). The top 252 OTUs with ρ > 0.7, *P* < 0.001 of Spearman correlation values were used for the network analysis (Supplementary Tables 3, 4). Overall, OTU numbers that satisfied the threshold ranged from 164 to 207 in the fruiting body and 155 to 200 in the dead wood (Supplementary Table 3).

**Figure 7:**
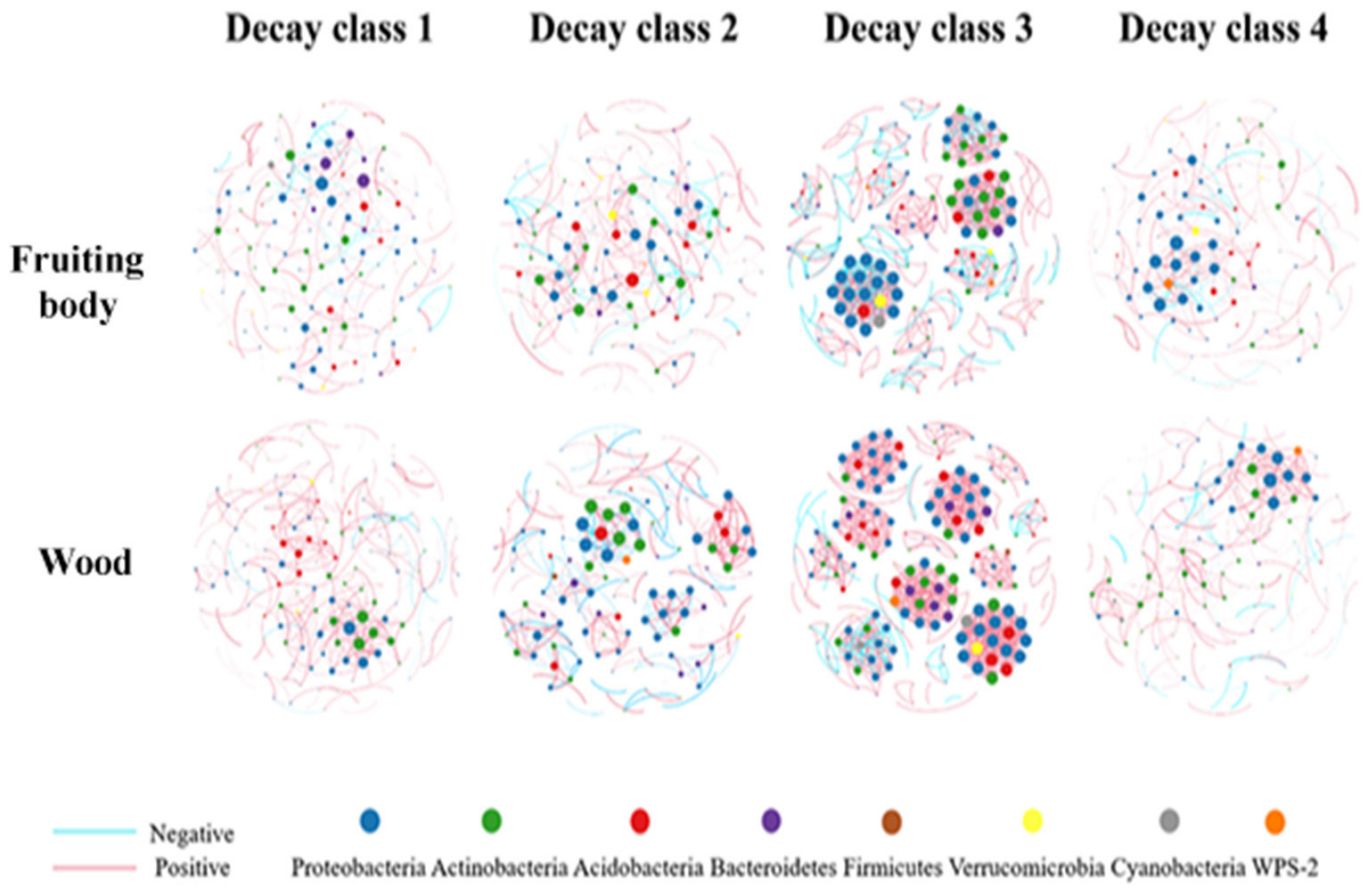
Co-occurrence networks of top 252 OTUs showing the significant correlations in fruiting body and wood over decay. Nodes represent individual OTUs; edges represent significant Spearman correlations (ρ > 0.7, *P* < 0.001). The size of node represents its link degree, the larger node has more connections with other nodes. The thickness of edge represents the correlation level. The red and blue edges show the positive and negative connections between nodes separately.

The connectivity in both substrates had the changing pattern of increasing before the third decay stage and decreasing at the fourth stage. The third decay class had the peak connectivity in both substrates, the lowest point was observed in D1F and D4W (Table 2; Figure 7). Connectivity in wood was higher than in the fruiting body in all the decay classes, which means that bacteria hosted by the decayed wood were more actively interacting with each other (Hartman et al., 2018). The modularity had a pattern of decrease-increase-decrease in the fruiting body, where D3F reached the peak and D4F was the lowest. In wood, the modularity had the opposite pattern, where D2W had the highest value, and D4W was the lowest (Table 2). The value of modularity indicated the modularization of community, which could be related to their function (Newman, 2006) and stability (Paranyushkin, 2012b).

**Table 2:** Detailed network properties of study groups.

**Group**	**OTUs**	**Connections**	**Connectivity**	**Po/Ne**	**Modularity**	**keystone**		
						**No**.	**Phylum**	**OTU ID**
D1F	164	219	2.67	6.87	0.81	2	Proteobacteria	6319
							Bacteroidetes	3156
D2F	165	283	3.43	5.29	0.75	1	Acidobacteria	254
D3F	207	619	5.98	2.89	0.88	18	Proteobacteria	26, 1, 13, 71, 251, 1121, 2914, 209, 67, 182, 220, 84, 6319, 5730, 811
							Cyanobacteria	7105
							Acidobacteria	36
							Verrucomicrobia	6984
D4F	146	245	3.36	5.45	0.74	1	Proteobacteria	57
D1W	189	333	3.52	22.81	0.79	1	Proteobacteria	54
D2W	159	303	3.81	1.97	0.89	6	Proteobacteria	2338, 7324, 31
							Actinobacteria	3094, 3544
							Acidobacteria	6429
D3W	200	833	8.33	15.03	0.88	19	Proteobacteria	5754, 6, 47, 431, 72, 209, 4967, 6196, 65, 375, 180, 5160
							Actinobacteria	6433, 124
							Acidobacteria	21, 36, 3738
							Cyanobacteria	18
							Verrucomicrobia	554
D4W	155	261	3.37	9.87	0.71	1	Proteobacteria	6517

*OTUs: Number of network nodes*.

*Connections: Number of network edges*.

*Connectivity: Mean number of connections per node*.

*Keystone OTUs: Selected based on the top 1% of node degree values of each network. Po/Ne: The positive and negative connecting ratio*.

The positive and negative connecting ratio (Po/Ne) used to represent connection stability (de Vries et al., 2018) had the opposite changing pattern to their modularity in both substrates. D3F and D2W had the minimum value, and D1F and D1W reached the peak (Table 2; Figure 7). Our result indicated that the communities in the fruiting body were more stable compared with wood as their Po/Ne values had smaller changing range (Table 2). Moreover, the thicker linking edges in the middle decay classes in both substrates also showed stronger connections (Figure 7). We selected nodes with top 1% connectivity in each group as the keystone OTUs (Hartman et al., 2018). We found that phylum Proteobacteria existed in all networks except D2F and was the only phylum that existed at the end of the decay process (Table 2). Among all selected OTUs, 11 of them were in top 50 abundant group; only OTU6319 reoccurred in D1F and D3F; OTU209 appeared in D3F and D3W; and all the rest were unique (Table 2).

## Discussion

### The Bacterial Communities of Fruiting Body and Associated Wood of Different Decay Classes

The alpha diversity and beta diversity of bacterial community did not show any significant differences neither in wood tissue nor in fruiting body. However, community structures, activeness, stabilities, and functions kept changing. At the end of the fourth decay process, new bacterial communities replaced the old ones previously constructed in living standing tree. Changes from host chemical-physical properties (Pent et al., 2020), interactions between hosts (Steffan et al., 2020) and microbiome (Bastías et al., 2020) might be the key parameters contributing to the bacterial community restructuring. This could be partly because the environmental factors such as soil property, moisture, and temperature were quite similar in the fruiting body as well as its adhering wood tissue.

At the first decay stage, bacterial biota in the fruiting body were more abundant, unique, active, and functional, however, the opposite trend was observed in the wood tissue. We assumed that in standing living tree, bacterial community of the fruiting body associated to it may have been suppressed, compared with dead wood tissue. However, the effect and trend weakened with progress of decay. It is very probable that the active defense system and associated chemical compounds in living trees (Asiegbu et al., 2005; Arnerup et al., 2010) may have contributed to the migration of the bacteria to the fruiting body. It is also likely that in decay class 1, bacteria were able to metabolize easily degradable sugars and carbohydrates, as well as utilizing the opportunity to source for nutrients directly from woody tissues (Clausen, 1996). Ray cell, which provides this kind of nutrients, was considered to be partly decomposed during this stage. Additionally, the increasing permeability of cellulosic substrates provides more chance for lateral bacterial groups to invade (Greaves, 1971). At the second decay class, a considerable number of bacteria groups dead from the first decay class serves as an important nitrogen resource, which lack in wood tissue. With further progress of the decay, more nitrogen and carbon resources become available (Greaves, 1971; King and White, 1976). Based on these, increasing number of nitrogen fixing bacteria start to be active with a better niche and able to have access to more resources (Johnston et al., 2016). In contrast to the living wood, plant endophytes and mycorrhiza helper bacteria (Lehr et al., 2007; Terhonen et al., 2016; Lipps and Samac, 2021) have been reported to support diverse microbial communities in that niche.

The third decay class was an important turning point for the whole decay process where diversity indicators, OTUs structures, the network topological parameters reached the extreme points, and functional groups started to be differentiated between the two study materials. According to previous studies (Mäkinen et al., 2006), the wood density and stem mass start to have a sharp decrease for conifer tissues at this stage. The physical status of deadwood directly changes the accessible oxygen for wood inhabiting microbiome. The aerobic bacteria have better chance to thrive, while the anaerobic bacteria are much more at a disadvantage in the community. It is therefore possible that at this decay class, the community might reach the limitation range due to constraints of nutrition (Hacquard et al., 2015), host property (Swedjemark et al., 1998; Gohar et al., 2020), and environmental factors (Probst et al., 2018).

We observed a dramatic change in stability indicator (Po/Ne) between D2W and D3W. Besides the accumulated nutritional factors, external perturbations rather than the gradual microenvironmental changes could also be the possible reason. The previous study showed that though a higher modularity indicated a higher network stability when facing external perturbations, the network would be susceptible when targeted attack happens on community's key stone OTUs (Paranyushkin, 2012b) or by uncertain perturbations (Kitano, 2004). The obvious keystone OTUs change between D2W and D3W, and the activated Cyanobacteria and Verrucomicrobia in D3W support this concept.

In the last decay stage, as the nutrition from the old resources diminishes, a new successional group of bacteria community was observed. The new bacterial structures and functional groups were well shaped in both substrates. However, the increased instability (Po/Ne) may indicate a new community restructuring phase, as the decomposed wood turns into the soil organic layer, and soil properties take the dominant role to influence the microbial community (Fierer et al., 2007; Johnston et al., 2016; Uroz et al., 2016; Dong et al., 2021).

We also found several soil- or plant-dwelling bacteria on *Heterobasidion* fruiting body, *Bacillus* spp. (Kjeldgaard et al., 2019), for example. As discussed in previous studies, the fruiting body microbiome was also mobile as mobility is a significant microbial feature (Mitchell and Kogure, 2006; Son et al., 2015). It is most probable that bacteria might be moving from living wood tissue to the fruiting body attached or associated with it. This is reflected in the increased number of bacteria OTUs in the fruiting body. However, when the tree is dead, the active bacteria in the fruiting body start to invade dead wood with extended duration or progress of decay process. It is also possible that the low numbers of OTUs in living wood are due to the less nutritious properties of wood (Maurice et al., 2021). The higher community activity and decreasing OTUs abundance in wood might also imply that the community members were more stable in wood, making it difficult for new species to join the community. The predominant bacteria in wood might be highly selected because of a tougher niche due to various plant secondary metabolites (Garzoli et al., 2021).

In this study, the environmental factors had no significant impact on the decay classes (Pent et al., 2017).

### The Ecological Functions of Bacteria Biota in Fruiting Body and Dead Wood

The FAPROTEX analysis confirmed nutrition, energy, degradation, and plant pathogen-related functions of the bacterial community. Several dominant taxonomic groups or those that showed significant changes were also of interest, as their functions are closely related to the community structure (Fierer et al., 2007).

*Burkholderia* spp. was the most abundant phylum in both substrates and was active in all decay classes (Figure 4; Supplementary Table 3). The abundance decreased with decay process in wood, though it was more consistent in the fruiting body. This result is similar to a previous observation on bacteria biota of the decayed wood (Probst et al., 2018). The genus' typical characteristic of being a fungal symbiont with associated fungal host protecting capability might explain its consistence in the fruiting body (Partida-Martinez and Hertweck, 2005; Marupakula et al., 2016; Sharmin et al., 2018). Furthermore, its general functions as nitrogen fixing (Sun et al., 2014), cellulose, and aromatic degrading (Kielak et al., 2016) made it lose competitive advantage in wood.

Firmicutes was much more abundant in wood tissue than in fruiting body across all decay classes and was significantly abundant in D3W. This result was similar to previous studies, where Firmicutes takes less proportion in mycorrhizosphere but is abundant in soil and wood tissue (Vik et al., 2013; Timonen et al., 2017). This might be because bacteria from this phylum have strong resilient capability in anaerobic condition (Hartmann et al., 2014). Studies from Shen et al. (2015) showed that enrichment of Firmicutes (*Bacillus*) suppresses Fusarium wilt disease. Studies by Trivedi et al. (2017) and Xiong et al. (2017) also indicate that Firmicutes is more abundant in disease-suppressed soil. Interestingly, this phylum acted non-remarkably in all decay stages and were mostly interacting within the phylum (Supplementary Figure 4). It is very probable that its powerful pathogen-suppressing capability contributed to its high stability and abundance.

Cyanobacteria and Verrucomicrobia only appeared in the third decay class in both substrates and only co-existed within the same module (Figure 7). Environmental factors were shown to be the primary driver for cyanobacterial community structuring (Rigonato et al., 2012). The decayed wood tissue might be the trigger driving the community restructuring as well as the secondary metabolites produced by Cyanobateria could suppress the pathogenic activity (Suurnäkki, 2015). Verrucomicrobia was proved to be an important phylum for plant health (Buée et al., 2009; Da Rocha et al., 2013), nutrient availability is the key factor for verrucomicrobial assemblage (Da Rocha et al., 2013; Uroz et al., 2013). Cyanobacteria provided nutrition by fixing nitrogen and carbon (Rigonato et al., 2012), which was also crucial for the activeness of both groups.

*Pseudomonas* spp. was abundant in both substrates in the first decay class; however, it decreased dramatically with progress of wood decay (Figure 4; Supplementary Tables 3, 4). Its ecological function might be different in the two substrates and in the different decay classes. In woody tissue, it could be endobacteria in a living tree (GŽibovska, 2016). The tree might also have supported its existence, but the beneficial interaction stopped when the tree was killed. Consequently, the genus had difficulty to survive on dead wood tissue (Pellicciaro et al., 2021). In the fruiting body, *Pseudomonas* decreased in relative abundance at the start of decay, which later increased considerably. Some species in this genus are also known to be opportunistic pathogens (Lipps and Samac, 2021). It is likely that *Pseudomonas viridiflava*, identified in this study, could be a pathogen, endophyte, or saprotroph (Lipps and Samac, 2021). The species might live as an endophyte when tree was alive but shifted to pathogenic behavior triggered by bacterial competition (Lipps and Samac, 2021). With no suppression from tree resistance, resulting in its increased abundance (Supplementary Table 3).

### The Network Analysis Advantages in Microbial Community Study

Microbiome analysis of *Heterobasidion* associated organisms has been widely conducted on the group level (e.g., fungi, bacteria, or virus) and specific species level; however, not much has been explored at the network level. Network metrics in this study indicated more detailed ecological (Bascompte, 2010; Thébault and Fontaine, 2010) and evolutionary (Olesen et al., 2007) information. The changing characteristics of the community were evident in this study. To our knowledge, this is the first study on the network analysis of bacteria from *Heterobasidion* fruiting body and its associated decaying wood. Our study also revealed a more complete changing trend of important bacteria throughout the various decay stages. However, the participation of fungi in both substrates merits to be investigated as it might provide further useful insight on the community structure, functions, and interaction with bacteria as well as the complete microbiome picture.

## Conclusion

The bacterial communities inhabiting the fruiting body and wood were found to be highly dynamic through the entire wood decay process. Physical-chemical properties of fruiting body and wood, properties of bacteria species, and the interactions among fruiting body-wood-bacteria might probably have had influence in the community assembly. The third decay class was an important shifting point for both study materials as reflected by network metrics and functional groups' differentiation. The bacterial community assembled in the course of the decay process in both fruiting body and wood tend to start a new round of successional changes reflecting the transition from living tree to dead wood and *vice versa*.

## Data Availability Statement

The datasets presented in this study can be found in online repositories. The names of the repository/repositories and accession number(s) can be found in the article/Supplementary Material.

## Author Contributions

FA and WR designed the study. RP, RK, and WR collected samples in the field. WR performed the experiments, analyzed the data, and wrote the manuscript. FA conceived the study and contributed in drafting the manuscript. All authors edited and approved the manuscript.

## Funding

This study was supported by Ministry of Agriculture and Forestry (Grant number 4400T-2002). This study was also supported by the Chinese Scholarship Council Doctoral Stipendium.

## Conflict of Interest

The authors declare that the research was conducted in the absence of any commercial or financial relationships that could be construed as a potential conflict of interest.

## Publisher's Note

All claims expressed in this article are solely those of the authors and do not necessarily represent those of their affiliated organizations, or those of the publisher, the editors and the reviewers. Any product that may be evaluated in this article, or claim that may be made by its manufacturer, is not guaranteed or endorsed by the publisher.

## Supplementary Material

Supplementary Figure 1 Rarefaction curve of study groups showing the sequence depth.

Supplementary Figure 2 PCoA analysis of bacterial communities in four decay stages in fruiting body.

Supplementary Figure 3 PCoA analysis of bacterial communities in four decay stages in wood tissue.

Supplementary Figure 4 Co-occurrence networks of Firmicutes from top 252 OTUs in fruiting body and wood over decay.

Supplementary Table 1 The basic information of each sampling point.

Supplementary Table 2 The relative abundance of functional groups.

Supplementary Table 3 The basic information of OTUs Node in the networks.

Supplementary Table 4 The basic information of co-occurrence edge in networks.
