# Supplementary figures and images for: Bacteria Community Inhabiting Heterobasidion Fruiting Body and Associated Wood of Different Decay Classes
Source: Front Microbiol. 2022 May 3;13:864619. doi: 10.3389/fmicb.2022.864619 (PMC9111749; doi:10.3389/fmicb.2022.864619)

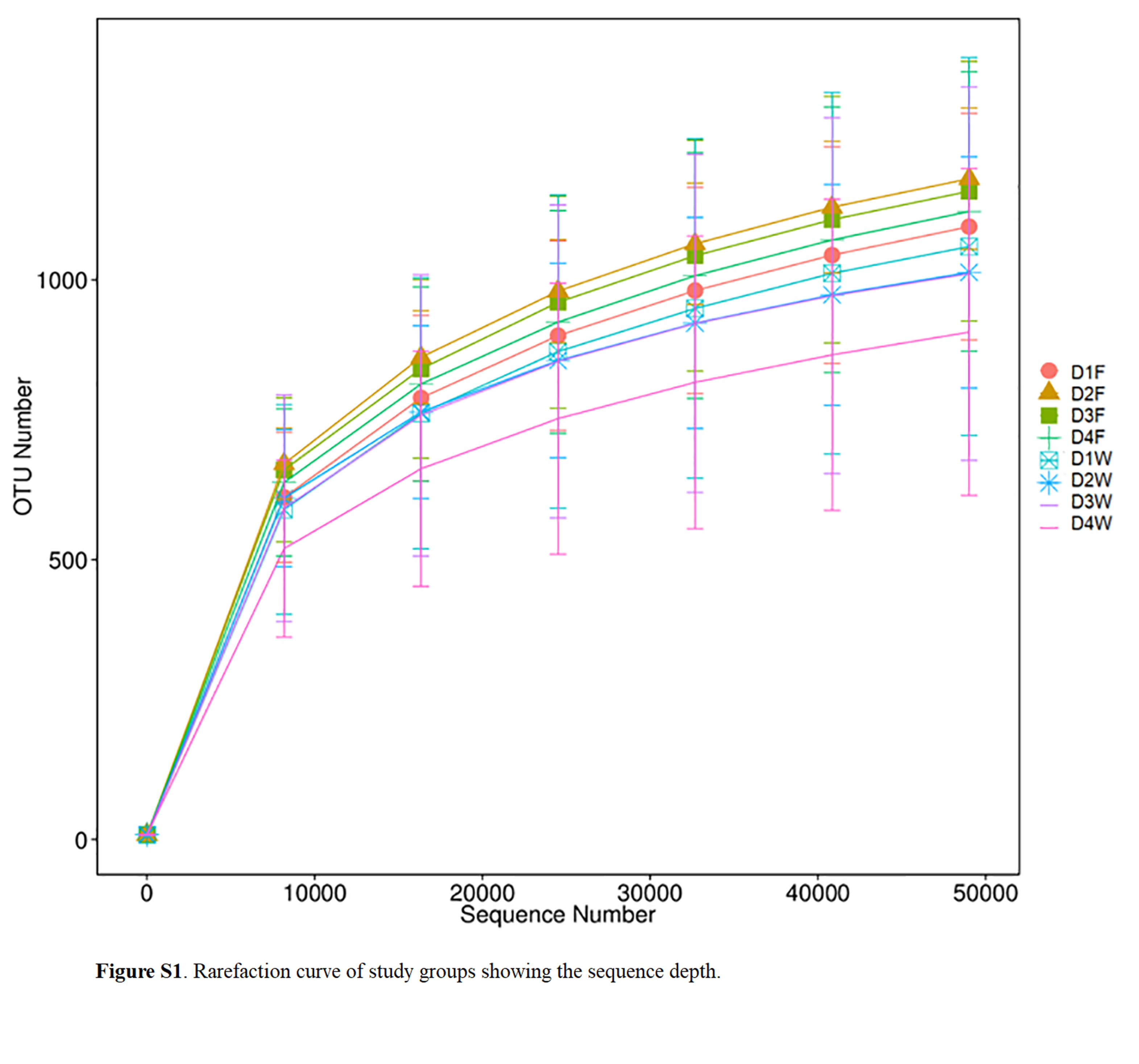

Supplement: Supplementary Figure 1 — Rarefaction curve of study groups showing the sequence depth. [file Image_1.tif]

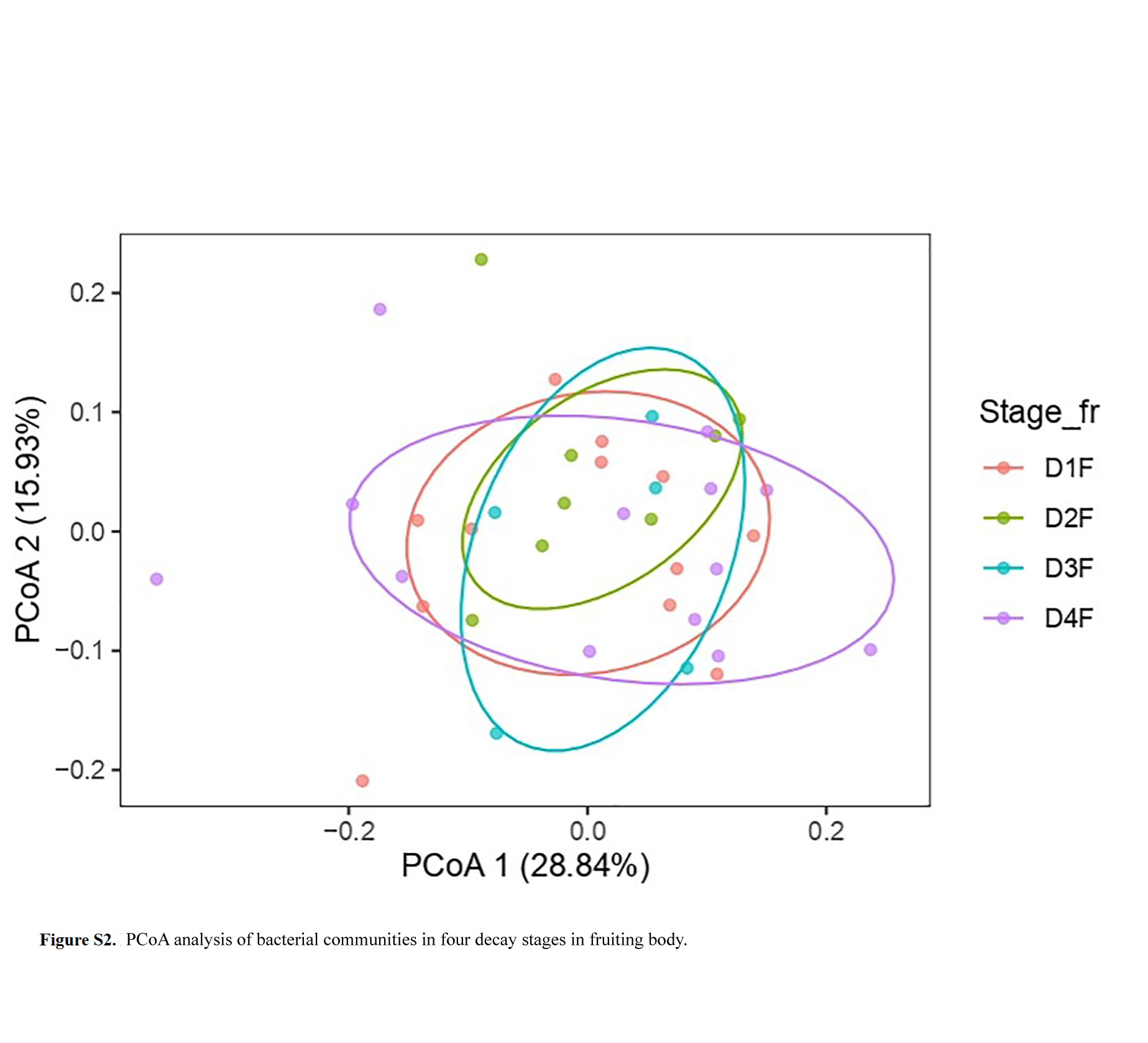

Supplement: Supplementary Figure 2 — PCoA analysis of bacterial communities in four decay stages in fruiting body. [file Image_2.tif]

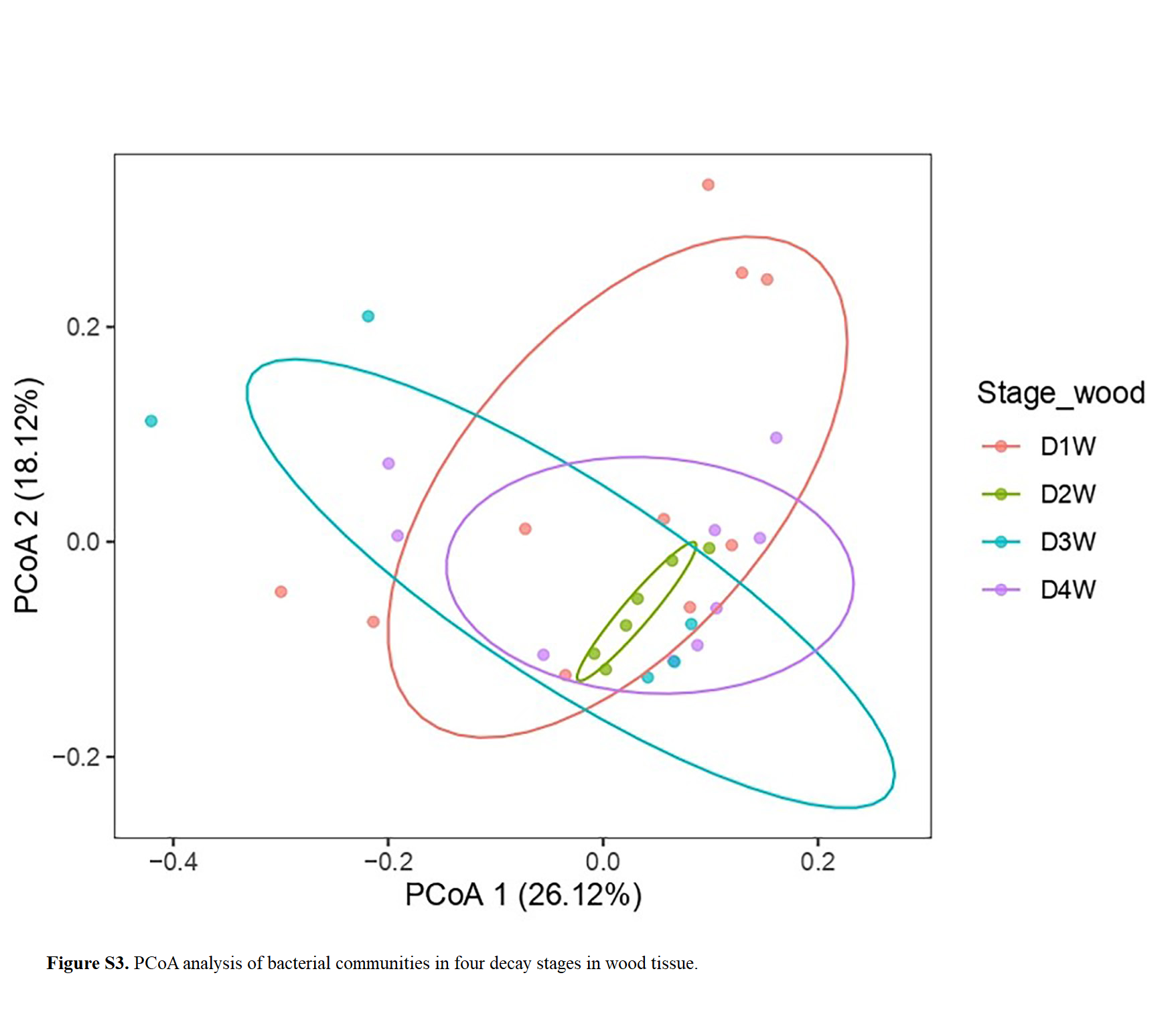

Supplement: Supplementary Figure 3 — PCoA analysis of bacterial communities in four decay stages in wood tissue. [file Image_3.tif]

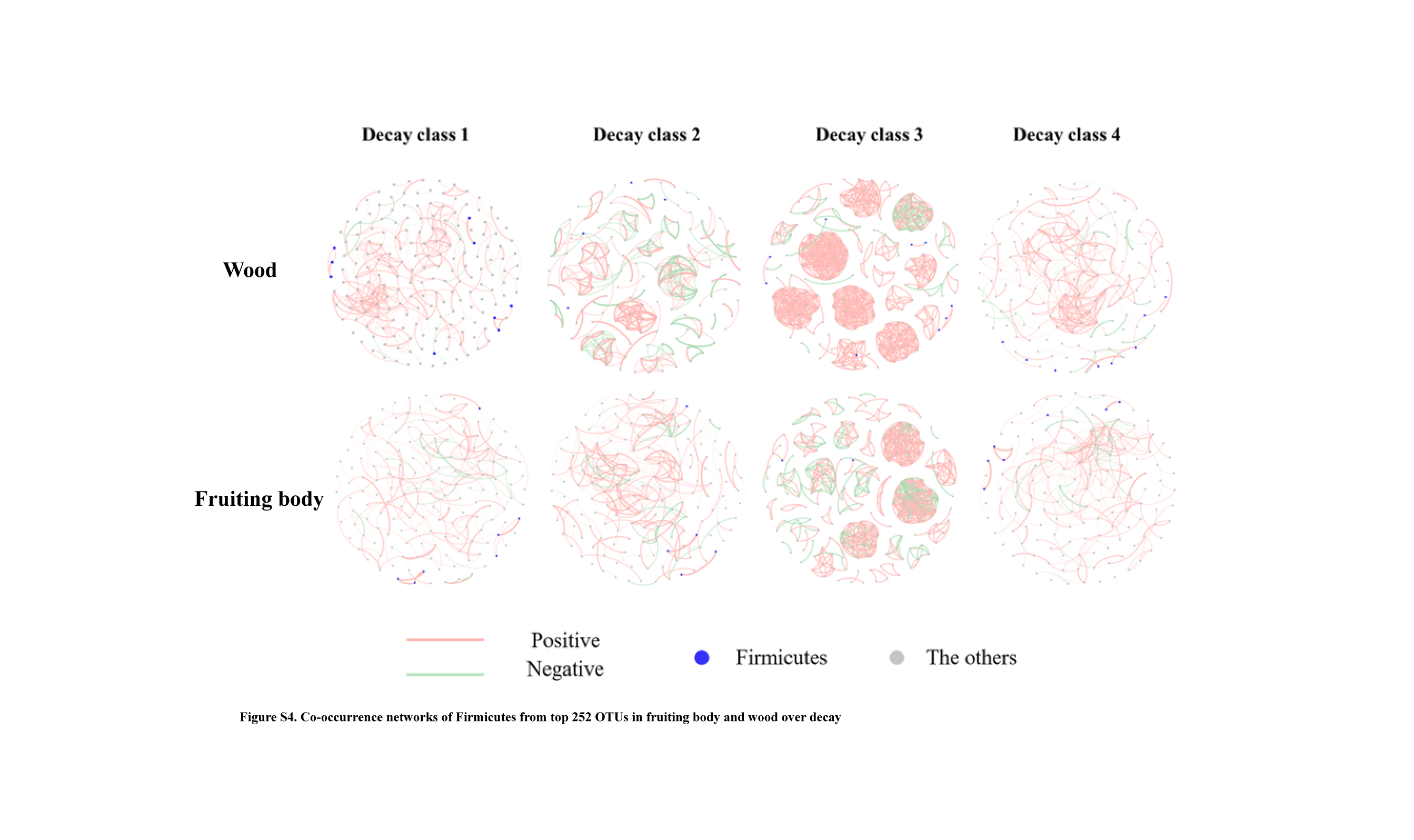

Supplement: Supplementary Figure 4 — Co-occurrence networks of Firmicutes from top 252 OTUs in fruiting body and wood over decay. [file Image_4.tif]
